# Incidence of sinus membrane perforation in transcrestal graftless maxillary sinus augmentation: a meta-analysis

**DOI:** 10.2340/aos.v85.46021

**Published:** 2026-06-04

**Authors:** Zhi-Cheng Huang, Xiao-Ling Wang, Qi-Qi Jin, Meng-Li Chen, Ya-Qin Zhao

**Affiliations:** a Department of Prosthodontics, Shaoxing Stomatological Hospital, Shaoxing City, China; b Department of Orthodontics, Shaoxing Stomatological Hospital, Shaoxing City, China; c Department of Preventive Dentistry, Shaoxing Stomatological Hospital, Shaoxing City, China

**Keywords:** sinus floor elevation, Schneiderian membrane perforation, graftless, osseodensification, osteotome, dental implant, meta-analysis, complication

## Abstract

**Objectives:**

This meta-analysis aimed to evaluate Schneiderian membrane perforation incidence and complications in graftless transcrestal sinus floor elevation.

**Materials and methods:**

Several databases (PubMed, Embase, Cochrane Library, Web of Science and Scopus) were searched for studies reporting perforation rates, early implant failure (≤12 months) and postoperative complications. Two reviewers independently selected studies, extracted data and assessed bias. Random-effects meta-analysis was performed. Evidence certainty was graded using the GRADE system.

**Results:**

Among 14 studies comprising 2119 sinus-lift sites, the pooled incidence of Schneiderian membrane perforation was 1% (95% CI: 0% (95% CI: 0% the pooled incidence of Schneiderian membrane perforation was 1% (95% CI: 0% months) and postoperative failure (≤12 months) was also 1% (95% CI: 0–6%) based on 711 evaluated implants, showing no heterogeneity (*I*² = 0%). No postoperative complications (acute sinusitis or epistaxis) were reported in the included literature. Subgroup analysis revealed that motor-driven techniques were associated with a significantly higher perforation rate (34%) compared to conventional osteotome methods (0%). The certainty of evidence was rated as very low for all outcomes, including membrane perforation and secondary indicators.

**Conclusions:**

Graftless transcrestal sinus elevation is associated with low Schneiderian membrane perforation rates, favourable implant survival and minimal postoperative complications. Despite methodological heterogeneity, current evidence supports the clinical safety of graftless techniques.

**Clinical relevance:**

Clinicians can confidently utilise graftless sinus floor elevation approaches with appropriate patient selection and careful surgical techniques to minimise complications.

## Introduction

Maxillary sinus floor elevation is a well-established surgical procedure widely used to facilitate implant placement in the posterior maxillae with insufficient residual bone height (RBH) [1, 2]. Initially described by Tatum and subsequently modified by Boyne and James, sinus-lift techniques have traditionally involved grafting materials introduced through a lateral window or crestal approach to augment bone volume for successful dental implant integration [3, 4]. Despite predictable clinical outcomes, traditional graft-based methods have several limitations, including prolonged healing times, higher treatment costs, and the potential risk of donor-site complications or biomaterial-related reactions, which may contribute to increased patient morbidity in some cases [5, 6].

To overcome these limitations, graftless transcrestal sinus elevation techniques have gained increasing attention due to their minimally invasive nature, reduced treatment time, cost-effectiveness and potential for accelerated healing [7]. Common graftless methods, such as osteotome sinus floor elevation (OSFE), osseodensification transcrestal sinus floor elevation (OD-TSFE) and minimally invasive sinus elevation (MISE), rely on controlled surgical elevation of the Schneiderian membrane to allow natural bone formation without adding external grafting materials. Several clinical studies have reported encouraging outcomes with these graftless techniques, demonstrating favourable implant survival rates and minimal postoperative complications [8–12].

However, one of the primary intraoperative concerns associated with sinus-lift procedures is Schneiderian membrane perforation, which can negatively influence implant stability and increase the likelihood of postoperative complications, such as acute sinusitis, implant failure or bleeding (epistaxis) [10, 13]. Previous systematic reviews have predominantly focused on traditional graft-based methods, with relatively limited and inconsistent reporting of membrane perforation and associated outcomes in graftless procedures [5, 14]. Additionally, variations in surgical techniques, instrumentation and clinical settings contribute to conflicting data regarding complication rates.

Therefore, the primary objective of this systematic review and meta-analysis is to evaluate the overall incidence of Schneiderian membrane perforation associated with graftless transcrestal sinus elevation procedures. Secondary objectives include assessing early implant failure rates (≤ 12 months postoperatively) and postoperative complications (acute sinusitis and epistaxis) to comprehensively inform clinical practice. We aim to synthesise existing evidence critically and quantitatively to provide robust clinical recommendations and to identify factors potentially influencing complication rates and clinical outcomes.

## Methods

### Eligibility criteria

Studies were eligible for inclusion if they met the following criteria:

Population: Adult patients undergoing graftless transcrestal maxillary sinus floor elevation procedures.

Intervention: Graftless transcrestal sinus floor elevation (e.g. OSFE, OD-TSFE, minimally invasive techniques).

Comparison: Not restricted; comparative and single-arm studies included.

Outcomes: Primary outcome is incidence of Schneiderian membrane perforation; secondary outcomes are early implant failure (≤12 months) and postoperative complications (acute sinusitis, epistaxis).

Study design: Randomised controlled trials (RCTs), prospective and retrospective cohort studies published in English.

Studies were excluded if they involved bone grafting, sinus-lift procedures via lateral window approach, animal or cadaveric studies, paediatric or mixed populations, or studies reporting fewer than 10 sinus-lift sites.

The unit of analysis was the site (bilateral cases counted as two sites). In multi-arm studies that included both grafted and graftless groups, only graftless arms were extracted; studies that could not be separated accordingly were excluded.

### Information sources and search strategy

A comprehensive electronic literature search was conducted in PubMed, Embase, Cochrane Library, Web of Science and Scopus databases between database inception and May 2025. The search combined controlled vocabulary (MeSH terms) and free-text keywords: (‘sinus floor elevation’ OR ‘sinus lift’ OR ‘crestal sinus elevation’ OR ‘osteotome sinus elevation’ OR ‘osseodensification’ OR ‘otranscrestal’) AND (‘membrane perforation’ OR ‘complication’ OR ‘implant failure’ OR ND (‘membrane perforation’ OR ‘Schneiderian membranei’) AND (‘graftless’ OR ‘without graft’ OR ‘without graft’ut graft’t graft’plic) AND (ithout graft’ut graft’t graft’). Additionally, the reference lists of included articles and relevant systematic reviews were manually searched to identify additional studies. The detailed search strategies for each database are presented below.

In PubMed, the following Boolean search string was used in title/abstract fields:

(‘n Pub floor elevationollowing Booleft’ OR ‘crestal sinus elevationevatioosteotome sinus elevationelevatosseoden sification’sseodtranscrestal’) AND (estalcationevationBooleft’ OR ‘h string was used in title/abstract fields:gies for each databaSchneiderian membranei) AND (ngraftless’raftlesshout graft’ OR ‘non-grafted’ OR ‘without bone graft’) AND (sshout graft’ OR ‘non-grafted’ NOT (animal).

In Embase (via Ovid), the following query was applied:

((via Ovid), the followinti,ab OR b OR d), the ti,ab OR bcrestal sinus elevationevti,ab OR bosteotome sinus elevationevti,ab OR bosseodensification’sti,ab OR btranscrestal’rti,ab) AND (AND (cationationing queti,ab OR b OR D (cationati,ab OR b OR D (cationatioti,ab OR b OR D (cationationing quti,ab OR bSchneiderian membraneiati,ab) AND (Agraftless’rti,ab OR blessD (membranti,ab OR b OR D (membrti,ab OR b OR D (membraneoninti,ab) AND (AND (membti,ab OR b OR D (membraneoti,ab) NOT (animals)/lim.

In the Cochrane Library (CENTRAL), the strategy was as follows:

(‘sinus floor elevation’ OR ‘sinus lift’ OR ‘crestal sinus elevation’ OR ‘osteotome sinus elevation’ OR ‘osseodensification’ OR ‘transcrestal’) AND (ANDcrestal’estal’ OR ‘transcrestal’ion’ OR ‘transcrestal’screstal’ion’ OR ‘transcrestal’resSchneiderian membranei) AND (graftless OR tless OR eaft’ OR ‘non-grafted’ OR ‘without bone graft’) AND (less OR eaft’ OR ‘non-grafted’:ti,ab,kw

In Web of Science Core Collection, we searched within topic fields (TS):

TS = (‘sinus floor elevation’ OR ‘sinus lift’ OR ‘crestal sinus elevation’ OR ‘osteotome sinus elevation’ OR ‘osseodensification’ OR ‘transcrestal’) AND TS = ( AND TS = al’estal’ OR ‘transcrestal’e lift’ OR ‘crestal sinus elevation’ OR ‘osteotome ’resSchneiderian membranei) AND TS = (graftless OR tless OR embraneR ‘transcrestal’e lift’ OR bone graft’) AND TS = (OR embraneR ‘transcrestal’e l.

In terms of filters, document type was limited to articles or reviews, and language was restricted to English.

In Scopus, the following string was used in title, abstract and keywords:

TITLE-ABS-KEY (‘sinus floor elevation’ OR ‘sinus lift’ OR ‘crestal sinus elevation’ OR ‘osteotome sinus elevation’ OR ‘osseodensification’ OR ‘transcrestal’) AND TITLE-ABS- KEY (OR ‘transcrestal’ion’ OR ‘transcrestal’screstal’ion’ OR ‘transcrestal’restal’d to English.esSchneiderian membranei) AND TITLE-ABS-KEY(graftless OR tless OR BS-KEY(graftless OR tal’ion’ OR ‘transcrestal’s) AND TITLE-ABS-KEY(Y(graftless OR tal’ion’ OR ‘tra (LIMIT-TO (LANGUAGE, ‘English’).

### Study selection

**Figure 1:**
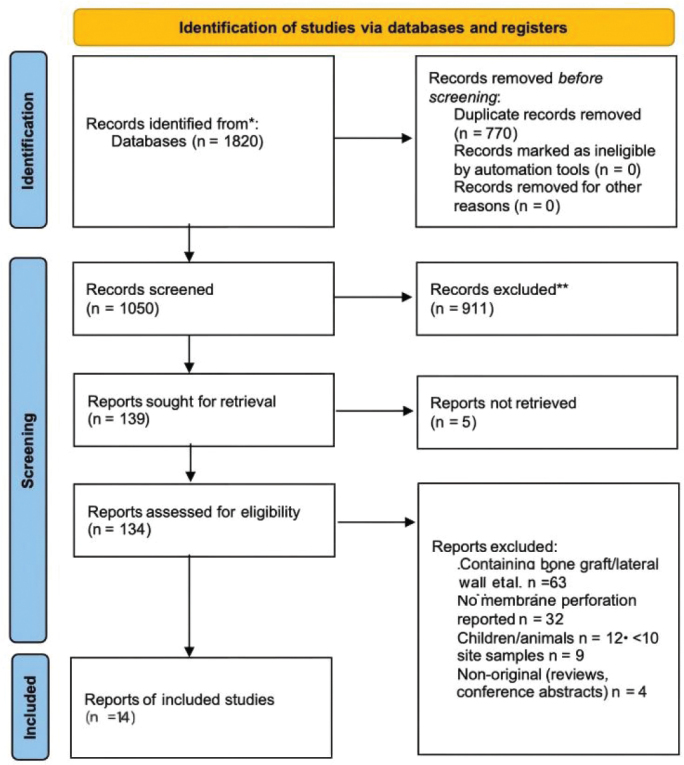
PRISMA 2020 flow diagram.

Two reviewers independently screened titles and abstracts against the eligibility criteria, followed by a full-text review of potentially eligible studies. Any disagreements were resolved by consensus or consultation with a third reviewer. Study selection https://doi.org/10.2340/aos.v85.46021 was documented using a PRISMA flow diagram (Figure 1).

### Data extraction

Two reviewers independently extracted data using a standardised extraction form, including the following aspects.

Study characteristics included first author, publication year, country, study design and sample size.

Patient and intervention characteristics included patient demographics, sinus-lift technique, implant placement and RBH.

Outcomes included the number of sinus-lift sites, events of Schneiderian membrane perforation, early implant failure events, postoperative complications and follow-up duration. Early implant failure was defined as loss or removal of an implant, or clinically detectable mobility occurring within 12 months after placement. Postoperative complications in this review focused on acute sinusitis and nasal bleeding (epistaxis). Acute sinusitis was defined as new-onset sinonasal symptoms (e.g. facial pain or pressure, nasal obstruction, purulent nasal discharge) with or without endoscopic or radiographic confirmation occurring after sinus floor elevation and requiring medical or surgical management. Nasal bleeding (epistaxis) was defined as postoperative bleeding from the nasal cavity attributed to the sinus-lift procedure and documented in the clinical record, irrespective of whether local haemostatic measures or additional treatment were necessary.

For unit and arm handling, we extracted outcomes by site. For studies reporting both grafted and graftless OSFE, we only extracted the graftless arm. When bilateral procedures were reported, each side was counted as an individual site. Studies that could not be disaggregated to graftless sites were excluded from quantitative synthesis. For studies reporting only mean RBH, we classified them overall into three groups based on the mean: RBH <4 mm, 4–6 mm and >6 mm.

Discrepancies were resolved by discussion and consensus.

### Risk of bias assessment

The risk of bias (RoB) for included studies was evaluated independently by two reviewers using the revised Cochrane RoB 2.0 tool for RCTs, covering randomisation, deviations from intended interventions, missing outcome data, outcome measurement and selective reporting. The ROBINS-I tool was used for non-randomised studies, assessing bias due to confounding, selection, intervention classification, deviations from interventions, missing data, outcome measurement and selective reporting.

Any discrepancies in the RoB assessment were resolved through consensus.

### Statistical analysis

Meta-analysis was performed using the R software with the meta package. Incidence data (perforation, implant failure, complications) were pooled using random-effects models with Freeman–Tukey double-arcsine transformation due to expected heterogeneity.

Heterogeneity was quantified using Cochran’s Q test and the I² statistic (with *I*² > 50% considered substantial).

Subgroup analyses were conducted based on study design (RCT vs non-RCT), surgical techniques and follow-up duration.

Meta-regression was conducted to investigate potential sources of heterogeneity, including RBH, surgical technique and year of publication.

Publication bias was assessed visually using funnel plots and statistically using Egger’s regression test.

Sensitivity analyses (including leave-one-out analyses) were performed to test the robustness of the results.

### Certainty of evidence

The certainty of the evidence for the primary and secondary outcomes was evaluated using the Grading of Recommendations Assessment, Development, and Evaluation (GRADE) framework, considering factors such as risk of bias, inconsistency, indirectness, imprecision and publication bias. Evidence was rated as high, medium, low or very low certainty.

Our GRADE assessment rated the certainty of evidence as medium for the primary outcome and low for secondary outcomes (early implant failure and postoperative complications). The medium certainty regarding membrane perforation incidence primarily reflects the large heterogeneity across studies, partly due to variability in surgical techniques, operator experience and patient characteristics. The low-certainty evidence for secondary outcomes arose from limited events, imprecision and indirectness of available data, underscoring the need for further high-quality studies with standardised outcome definitions.

## Results

### Study selection

Database searches yielded 1820 records. Following the removal of duplicates, 1050 titles/abstracts were screened. 134 full‐text articles were assessed for eligibility, and 14 original studies fulfilled the inclusion criteria (Figure 1). Reasons for exclusion at full-text stage were use of bone grafts (*n* = 63), no membrane perforation reported (*n* = 32), Children/animals (*n* = 12), sample size < 10 sites (*n* = 9) and Non-original (reviews included conference abstracts) (*n* = 4). The final pool comprised four RCTs and 10 non-randomised cohort studies, published between 2012 and 2024. The ≥10-site inclusion threshold was enforced at the level of the graftless analysis arm. Accordingly, Karaca was included because the study’s graftless OSFE arm met the ≥10-site threshold, even though the paper reports 14 patients; patient count was not the screening threshold.

### Study characteristics

Table 1 [7, 9, 15–26] summarises the principal characteristics of the 14 included studies. Altogether, the studies enrolled 1370 adult patients and 2119 crestal sinus-lift sites. Most studies used a classical osteotome technique (OSFE, 10/14); one employed OD-TSFE drills, two applied motor-driven or minimally invasive kits (MISE/Motor-driven OD), one applied Information Systems and Future Education (ISFE), and none adopted a mixed TSFE protocol following the exclusion of heterogeneous cohorts. Mean pre-operative RBH ranged from 2.4 to 7.4 mm; 28.5% of sites presented with RBH ≤ 5 mm. Follow-up varied from 0 months to 10 years (median: 24 months). The pooled intra-operative Schneiderian membrane perforation count was 95/2119 sites (4.48%) before quantitative synthesis. In multi-arm reports that included both grafted and graftless procedures, we only retained the graftless arm for all analyses.

**Table 1:** Baseline characteristics of the 18 included graft-less crestal sinus-lift studies.

Study (year)	Country	Design	Patients/Sites	Technique*	Mean RBH (mm)	Implant system	Diameter (mm)	Max follow-up	Perforations *n*/*N* (%)	Early implant failure rate	Bone_Graft
Nedir (2012) [15]	Switzerland	RCT	12/17	OSFE (8 mm)	2.6	Straumann	3.3–4.1	12 mo	0 /17 (0)	0 /17 (0)	No
Brizuela (2014) [16]	Spain	Prospective	37/36	OSFE	7.4	Nobel Biocare	3.75–4.3	24 mo	4/36 (11.1)	1/36 (2.8)	No
Marković (2015) [9]	Serbia/Spain	RCT	45/180	OSFE	6.6	Astra Tech	3.5–4.5	24 mo	0/180 (0)	0/180 (0)	No
Zill (2016) [17]	Germany	Retro.	113/233	OSFE	5.9	Straumann	3.3–4.8	60 mo	14/233 (6.0)	/	No
French (2016) [18]	Canada	Retro.	541/926	OSFE	–	Nobel Biocare	3.75–4.3	120 mo	1/926 (0.1)	/	No
Nedir (2016) [19]	Switzerland	RCT	12/17	OSFE (8 mm)	2.4	Straumann	3.3–4.1	60 mo	0/17 (0)	0/17 (0)	No
Si (2016) [20]	China	Retro.	80/96	OSFE	6.75	ITI/MIS	3.3–4.3	108 mo	0/96 (0)	7/96 (7.3)	No
Rawat (2019) [21]	India	Prospective	21/26	OSFE	6.8	Nobel Biocare	3.75–4.3	6 mo	1/26 (3.8)	/	No
Qian (2020) [22]	China	RCT	19/19	OSFE	4.58	ITI	3.3–4.3	120 mo	0/19 (0)	0/19 (0)	No
Rammelsberg (2020) [23]	Germany	Retro.	138/217	ISFE	6.8	Camlog	3.8–4.3	96 mo	47/217 (21.7)	8/217 (3.7)	No
Karaca (2022) [24]	Turkey	Prospective	14/14	OSFE	6.7	Nobel Biocare	3.75–4.3	6 mo	0/14 (0)	0/14 (0)	No
Alzoubi (2024) [25]	Kuwait	Prospective	29/29	Motor-OD	–	Densah burs/MIS implants	3.8–4.3	8 mo	10/29 (34.5)	3/29 (10.3)	No
Albadani (2024) [7]	Yemen	Prospective	60/60	MISE	6.88	Nobel Biocare	3.75–4.3	12 mo	0/60 (0)	0/60 (0)	No
Mazor (2024) [26]	Multi	Cross-section	249/249	OD-TSFE	5.4	Densah burs/various	3.5–4.5	Immediate	18/249 (7.2)	/	No

* OSFE: osteotome sinus floor elevation; OD-TSFE: osseodensification transcrestal sinus lift; Motor-OD: motor-driven crestal lift screw; MISE: minimally invasive sinus elevation; PRF: platelet-rich fibrin; RBH: residual bone height.

### Risk of bias assessment

Risk of bias was assessed separately for RCTs using the RoB 2.0 tool and for non-randomised studies using the ROBINS-I tool. All four included RCTs demonstrated an overall low risk of bias across domains, including randomisation, deviations from intended interventions, missing data, outcome measurement and reporting bias (Supplementary Table 1). Similarly, the 10 non-randomised studies were consistently judged as having a low risk of bias across all ROBINS-I domains, encompassing confounding, participant selection, classification of interventions, deviations, missing data, measurement of outcomes and reporting (Supplementary Table 2). Overall, these findings indicate robust methodological quality and reliability of the evidence base included in this meta-analysis.

### Results of meta-analysis of main and secondary indicators

**Figure 2:**
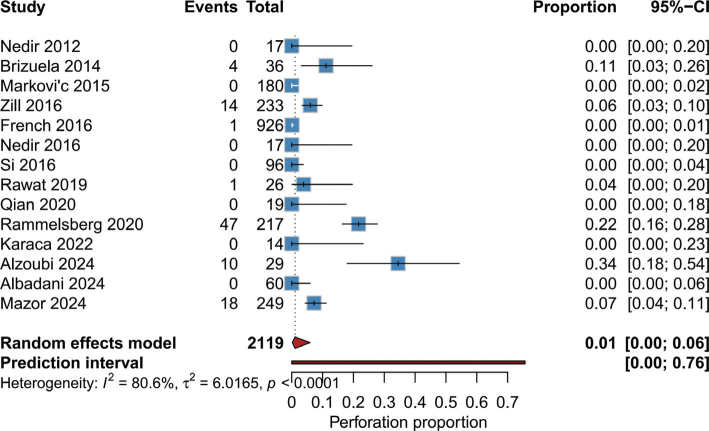
Forest plot of Schneiderian membrane perforation incidence.

Overall incidence of Schneiderian membrane perforation: The pooled random-effects analysis showed an overall Schneiderian membrane perforation incidence of 1% (95% CI: 0–96%) among the total 2119 sinus-lift sites from the 14 included studies (Figure 2). There was significant heterogeneity across studies (*I*² = 80.6%, τ² = 6.0165, *p* < 0.0001).

**Figure 3:**
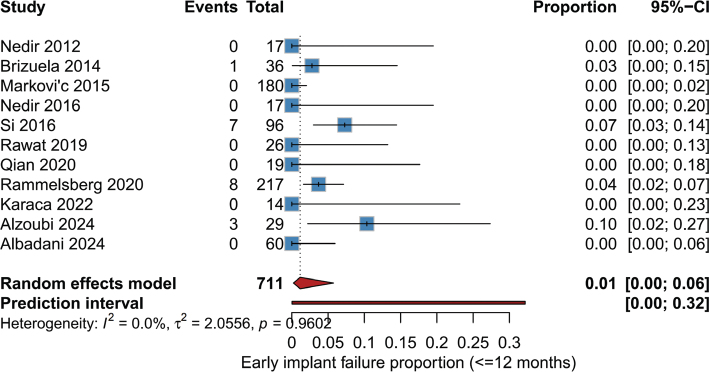
Forest plot of early implant failure rate (≤12 months).

Early implant failure rate (≤12 months): The random-effects meta-analysis of early implant failure (Figure 3) showed a pooled incidence of 1% (95% CI: 0–6%) among 711 implants evaluated. No heterogeneity was observed (*I*² = 0.0%, τ² = 2.0556, *p* = 0.9602). The highest failure rates were noted in the studies of Si [20] (7.3%), and Alzoubi [25] (10.3%), whereas most other studies reported low or zero events.

Regarding postoperative complications, no reports of such complications were found in the included literature (Supplementary Figure 1).

### Subgroup analyses

#### By study design

**Figure 4:**
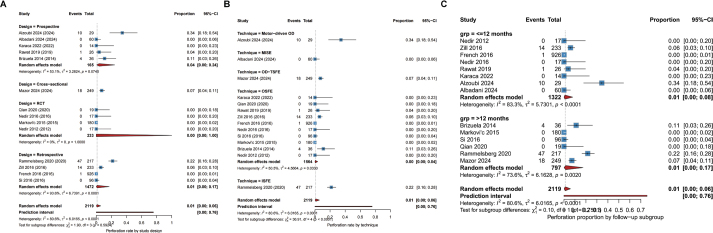
Forest plots of subgroup analyses for the incidence of Schneiderian membrane perforation. (A) Subgroup analysis by study design. (B) Subgroup analysis by surgical technique. (C) Subgroup analysis by follow-up duration.

When stratified by study design (Figure 4A), the pooled incidence of membrane perforation varied numerically but not statistically. Prospective studies reported a pooled rate of 4% (95% CI: 0–24%, *I*^2^ = 53.1%), whereas retrospective studies showed a pooled rate of 1% (95% CI: 0–17%, *I*^2^ = 93.6%). Notably, the four included RCTs reported zero perforation events (Pooled proportion: 0%; 95% CI: 0–1%, *I*^2^ = 0%). However, the test for subgroup differences indicated that study design was not a significant moderator of perforation risk (χ^2^ = 1.90, df = 3, *p* = 0.592).

#### By surgical technique

Subgroup analysis by surgical technique revealed significant variations in perforation rates (Figure 4B). The conventional OSFE technique demonstrated the lowest pooled perforation rate of 0% (95% CI: 0–4%, *I*^2^ = 50.5%). In contrast, studies utilizing Motor-driven OD reported a substantially higher pooled incidence of 34% (95% CI: 18–54%). The OD-TSFE and MISE techniques showed intermediate rates of 7 and 0%, respectively. Crucially, the difference between these technique subgroups was statistically significant (χ^2^ = 36.91, df = 4, *p* < 0.001), suggesting that the specific instrumentation and kinematics of the elevation method significantly influence membrane integrity.

#### By follow-up duration

To determine if the timing of outcome assessment influenced the reported perforation rates, we stratified studies into short-term (≤12 months) and long-term (>12 months) follow-up groups (Figure 4C). The pooled perforation rate was consistent across both timeframes: 1% (95% CI: 0–8%) for the short-term group and 1% (95% CI: 0.00–0.17) for the long-term group. No statistically significant difference was found between the two time points (χ^2^ = 0.10, df = 1, *p* = 0.750), indicating that the reporting of this intraoperative complication is not biased by the length of the postoperative observation period.

### Meta-regression

Initially, we assessed the impact of mean RBH as a continuous variable (Supplementary Figure 2A). The univariate analysis revealed no statistically significant association between RBH and perforation risk (β = −0.0064, *p* = 0.760). The model failed to explain any of the between-study variance (*R*^2^ = 0.0%), indicating that anatomical bone height alone is insufficient to predict perforation outcomes in this dataset. In contrast, when surgical technique was introduced as a categorical moderator (Supplementary Figure 2B), the model fit improved dramatically. The test for moderators was highly significant (Q_M_ = 20.99, df = 4, *p* = 0.0003), and the model successfully explained 66.9% of the total heterogeneity. Visually, distinct separation was observed between techniques, with Motor-driven OD associated with higher perforation estimates compared to MISE and OSFE. We further tested whether the effect of RBH persisted after controlling for surgical technique (Supplementary Figure 2C). In this adjusted model, the coefficient for RBH shifted to a positive direction (β = 0.0183) but remained statistically non-significant (*p* = 0.269). While this model explained a substantial amount of heterogeneity (*R*^2^ = 63.2%), it did not outperform the technique-only model. Finally, we compared the goodness-of-fit for all models using Akaike Information Criterion (AIC) and *R*^2^ values (Supplementary Figure 2D). Model B (Technique Categories) emerged as the optimal model, demonstrating the lowest AIC (–13.2) and the highest percentage of explained variance (66.9%). This confirms that surgical technique is the predominant factor influencing membrane perforation rates, overshadowing anatomical factors like RBH or temporal factors like publication year in our cohort of graftless sinus lift procedures.

**Figure 5:**
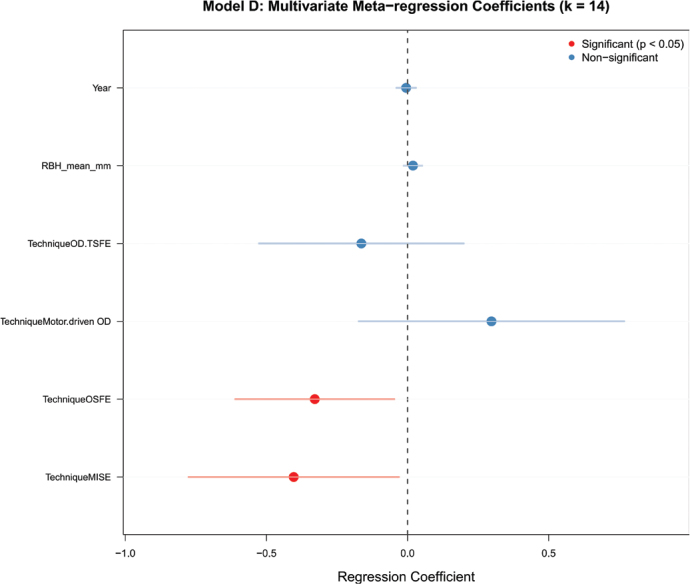
Multivariate meta-regression coefficients for predictors of membrane perforation.

To further investigate the sources of heterogeneity beyond subgroup analysis, we conducted a multivariate meta-regression including publication year, mean RBH, and surgical technique as covariates (Figure 5). The analysis identified surgical technique as a critical determinant of perforation risk. Specifically, both MISE and OSFE demonstrated statistically significant negative regression coefficients (*p* < 0.05, indicated by red dots), suggesting that these techniques are independently associated with a reduced risk of membrane perforation compared to other methods included in the reference category. Conversely, neither mean RBH nor publication year was found to be a significant moderator in this multivariate model (blue dots, *p* > 0.05), as their confidence intervals crossed the null value. Similarly, while numerical differences were observed, the coefficients for OD-TSFE and Motor-driven OD techniques did not reach statistical significance in this specific model configuration, likely due to the limited number of studies employing these newer protocols (*k* < 3 for some subgroups) and wide confidence intervals. These results reinforce that the choice of surgical instrumentation and kinematics (specifically, manual osteotomes or minimally invasive kits) is a more robust predictor of safety than anatomical factors like RBH alone in this dataset.

### Sensitivity analyses

To assess the robustness of our primary outcome, we performed a leave-one-out sensitivity analysis (Supplementary Figure 3). Sequentially removing each study from the analysis revealed that no single study exerted an undue influence on the overall pooled estimate. The pooled perforation rates remained stable across all iterations, ranging narrowly between 0.03 and 0.06 (3–6%), with overlapping confidence intervals. This consistency suggests that our findings are reliable and not driven by any single outlier or high-weight study.

Despite minor variations observed, the 95% CIs in all leave-one-out scenarios overlapped significantly, reinforcing confidence in the robustness and generalisability of the overall findings. Overall, no single study exerted a dominant influence, suggesting that the pooled results were not driven by any particular outlier or extreme-value study.

### Publication bias

Visual inspection of the funnel plots revealed distinct patterns across the different outcomes (Supplementary Figure 4). For the primary outcome of Schneiderian membrane perforation (Supplementary Figure 4A) and early implant failure (Supplementary Figure 4B), the distribution of studies appeared generally symmetrical around the pooled effect size. Egger’s linear regression test confirmed no statistically significant publication bias for these two outcomes (*p* > 0.05). Furthermore, the trim-and-fill method indicated that no imputed studies were required to correct for asymmetry in the perforation analysis, and the adjusted pooled rate remained consistent with the primary estimate. The 95% prediction interval suggested that future studies would likely observe perforation rates between 0 and 6%.

However, the funnel plot for overall complications (Supplementary Figure 4C) displayed evident asymmetry. There was a notable absence of small-sample studies reporting higher complication rates (left side of the plot), while studies reporting lower complication rates clustered tightly on the right. This skewness suggests potential publication bias, where smaller trials with unfavourable safety outcomes may be less likely to be published. Consequently, the pooled estimate for overall complications should be interpreted with caution, as the true incidence in broader clinical practice might be higher than reported in this selected literature.

### GRADE certainty of evidence

Using the GRADE approach, the certainty of evidence was rated as follows (Supplementary Table 3).

Schneiderian membrane perforation (primary outcome): Very Low, downgraded due to significant heterogeneity (*I*² = 80.6%).

Early implant failure (≤12 months): Very Low, downgraded for imprecision (few events, wide CIs) and indirectness (variable follow-up and definitions).

Postoperative complications (acute sinusitis/epistaxis): Very Low, downgraded due to imprecision (very few events) and indirectness (limited reporting, varying definitions).

## Discussion

This systematic review and meta-analysis investigated the incidence of Schneiderian membrane perforation and related complications following graftless transcrestal maxillary sinus floor elevation procedures. Our findings indicate an overall Schneiderian membrane perforation rate of 4.48%, highlighting the clinical acceptability and relative safety of graftless sinus-lift techniques. The present meta-analysis included 14 studies (4 RCTs and 10 non-randomised studies) involving a total of 2119 sinus-lift sites, providing a robust dataset to quantify risks associated with these widely used implant-placement techniques.

Crucially, beyond membrane thickness and bony septa, the transverse dimension of the maxillary sinus (sinus width) has emerged as a pivotal anatomical predictor of perforation risk in transcrestal approaches. Although our meta-regression did not identify RBH as a significant moderator, we must highlight the profound influence of sinus anatomy reported in recent large-scale observational studies. Specifically, Stacchi et al. [27] demonstrated a striking correlation between sinus width and membrane integrity, reporting that the incidence of perforations in wide sinuses was approximately 15 times higher than in narrow sinuses during transcrestal procedures. This dramatic increase is likely attributable to the biomechanical challenges inherent in wider anatomies: in narrow sinuses, the osteotome engages both lateral walls, facilitating the formation of a stable ‘bone plug’ that protects the membrane during elevation. Conversely, in wide sinuses, the lack of lateral wall contact prevents effective bone plug formation, requiring the surgeon to rely solely on apical pressure, which significantly increases the risk of uncontrolled membrane tearing. This finding underscores that RBH alone is insufficient for risk stratification; preoperative assessment of sinus width via cone beam computed tomography (CBCT) is essential. Clinicians should exercise heightened caution or consider alternative approaches (e.g. lateral window technique) when encountering significantly wide sinus anatomies, even if RBH appears favourable.

Our analysis showed significant heterogeneity across studies (*I*² = 80.6%) concerning the primary outcome of membrane perforation. Subgroup analysis and meta-regression indicated that surgical technique was the main contributor to this heterogeneity, whereas RBH showed no consistent or statistically significant effect. Notably, studies employing the classical OSFE technique generally demonstrated lower membrane perforation rates, whereas newer techniques such as motor-driven OD-TSFE showed numerically higher perforation rates in single-cohort studies, although these differences were not statistically significant after adjustment for technique category in meta-regression. This variation may relate to differences in operator experience, surgical instrumentation, sinus anatomy or technical sensitivity. Clinicians should carefully evaluate technique selection, balancing the clinical advantages of newer techniques with their associated learning curves and complication risks [27, 28]. Li et al. [28] reported that membrane perforation increased early implant failure risk by more than threefold (OR = 3.41), particularly in sites with RBH ≤ 4 mm or where excessive osteotome pressure was applied. Nemati et al. [29] further demonstrated that membrane thickness < 1 mm, sharp bony septa and insufficient visibility during surgery significantly elevated perforation risk (ORs ranging 2.8–4.6). Together, these findings highlight that anatomical complexity and operator-dependent technique execution remain critical determinants of intraoperative safety in both crestal and lateral window sinus elevation procedures. In particular, motor-driven OD-TSFE is performed using high-speed counterclockwise rotation (1100 rpm) with vertical ‘bouncing’ movements for intraosseous expansion. Several reports have suggested that excessive rotational speed and torque may generate negative pressure or centrifugal forces near the sinus floor, increasing technical difficulty and predisposing to membrane tears. Moreover, this technique requires a considerable learning curve; inadequate operator training could be one reason for the markedly higher perforation rates observed. It is therefore recommended that clinicians strictly follow manufacturer guidelines for speed and torque control and receive adequate training before adopting this procedure in clinical practice.

Early implant failure (≤ 12 months) was another important outcome examined. The overall pooled early implant failure rate was 0%, which is clinically acceptable and aligns with previously published rates in traditional sinus augmentation with grafting. Most included studies reported extremely low failure rates, indicating overall implant stability in graftless sinus lifts [15, 16]. Nonetheless, higher early failures in individual studies appeared linked to the use of implants in sites with extremely limited RBH or complicated intraoperative membrane perforations [25, 30]. Careful patient selection, precise surgical technique and cautious postoperative management should be emphasised to further mitigate such early complications.

All the reports showed no complications. This low complication profile underlines the minimally invasive nature and inherent safety of graftless approaches, particularly when surgeons adhere strictly to standard operating protocols and rigorous patient monitoring [31, 32]. For example, Gao et al. retrospectively evaluated the cushioned grind-out technique using CBCT in patients with severely atrophic maxillae and found that RBH ≤ 4 mm did not increase the risk of membrane perforation or implant failure; the 7-year cumulative implant survival rates were 95.5 versus 93.9% [31].

Additional evidence also comes from recent observational studies. Yang et al. reported a perforation rate of 0.6% with a mean RBH of 4.2 mm in a retrospective series of robot-assisted transalveolar sinus floor elevation [10]. Karande et al. employed CBCT to compare Schneiderian membrane thickness between smokers and non-smokers, showing that smokers had significantly thicker membranes [13]. These findings highlight how emerging technologies and patient factors can influence procedural risks and outcomes.

The strengths of this systematic review and meta-analysis include the comprehensive literature search, careful RoB assessment (with all studies ultimately rated as low risk) and rigorous statistical methods to ensure robust conclusions. Limitations, however, exist, including inherent study heterogeneity, variable follow-up periods and inconsistencies in reporting secondary outcomes across included studies. Another limitation of our subgroup analyses concerns the categorisation of RBH. For several studies that reported only mean RBH values without stratified distributions, we assigned the entire cohort into a single RBH category (< 4 mm, 4–6 mm or > 6 mm) according to the reported mean. While this approach allowed for inclusion of more studies in the subgroup analyses, it inevitably carries the risk of misclassification at the individual level, as patients with RBH values near category thresholds may have been allocated differently if individual data were available. This limitation should be considered when interpreting subgroup comparisons and highlights the need for future studies to report more granular baseline RBH data to facilitate accurate stratified analyses. Further multicentre RCTs and large prospective cohort studies utilising standardised reporting frameworks are recommended to refine complication estimates and enhance clinical guidance.

## Conclusion

In conclusion, graft-free transcrestal maxillary sinus floor augmentation is associated with an acceptably low rate of Schneiderian membrane perforation and postoperative complications, as well as good early implant survival. Clinicians considering graft-free techniques can be confident in their safety, especially when appropriate patient selection and meticulous surgical technique are prioritised. Although current evidence suggests a favourable safety profile, long-term follow-up studies are needed to verify long-term outcomes. Furthermore, future studies should aim to optimise the consistency of patient selection criteria, surgical techniques and outcome measures to enhance future evidence and inform clinical practice guidelines.

## Supplementary Material





## Data Availability

Data sharing is not applicable to this article as no new data were created or analysed in this study.

## Declarations

Ethics approval and consent to participate.

Since this study is based on publicly available data, no ethical approval is required.
