# Supplementary material for: Incidence of sinus membrane perforation in transcrestal graftless maxillary sinus augmentation: a meta-analysis
Source: Acta Odontol Scand. 2026 Jun 4;85:46021. doi: 10.2340/aos.v85.46021 (PMC13241954; doi:10.2340/aos.v85.46021)
Supplement: Supplementary file 1 [file AOS-85-46021-s1.pdf]

Supplementary material has been published as submitted. It has not been copyedited or typeset by Acta Odontologica Scandinavica.

Supplementary Table 1 Risk of Bias for Randomized Controlled Trials (RoB 2.0)

Study	Randomization	Deviations from intervention	Missing data	Measurement	Selective reporting	Overall risk
Nedir 2012	Low	Low	Unknown	Low	Low	Low
Marković 2015	Low	Low	Low	Low	Low	Low
Nedir 2016	Low	Low	Unknown	Low	Low	Low
Qian 2020	Low	Low	Low	Low	Low	Low

Supplementary Table 2 Risk of Bias for Non-Randomized Studies (ROBINS-I)

**Study**	**Confounding**	**Selection**	**Classification**	**Deviations**	**Missing data**	**Measurement**	**Reporting**	**Overall risk**
Brizuela 2014	Low	Low	Low	Low	Low	Low	Low	Low
Zill 2016	Low	Unknown	Low	Low	Low	Low	Unknown	Low
French 2016	Unknown	Unknown	Low	Low	Unknown	Low	Low	Unknown
Si 2016	Low	Unknown	Low	Low	Low	Low	Low	Low
Rawat 2019	Unknown	Low	Low	Low	Low	Low	Unknown	Low
Rammelsberg 2020	Unknown	Low	Low	Low	Low	Low	Low	Low
Karaca 2022	Low	Unknown	Low	Low	Low	Low	Low	Low
Alzoubi 2024	Low	Low	Low	Low	Low	Low	Low	Low
Albadani 2024	Low	Unknown	Low	Low	Low	Low	Low	Low
Mazor 2024	Low	Low	Low	Low	Low	Low	Unknown	Low

Supplementary Table 3 GRADE Assessment of the Certainty of Evidence

**Outcome**	**No. of Studies**	**Sites (N)**	**Events**	**Pooled Proportion (95% CI)**	**Heterogeneity**	**Risk of Bias**	**Inconsistency**	**Indirectness**	**Imprecision**	**Publication Bias**	**Total Downgrades**	**Final Certainty**
Schneiderian membrane perforation	14	2119	95	1.09% (0.19% – 5.97%)	80.6%	Not downgraded	Downgraded (-1)	Not downgraded	Not downgraded	Downgraded (-1)	-2	Very low
Early implant failure <=12 months	11	711	19	1.15% (0.22% – 5.75%)	0.0%	Not downgraded	Not downgraded	Downgraded (-1)	Downgraded (-1)	Downgraded (-1)	-2	Very low
Post-operative complications (sinusitis/epistaxis)	14	2119	0	0% (0% – 1.0%)	0.0%	Not downgraded	Not downgraded	Downgraded (-1)	Downgraded (-1)	Not downgraded	-1	Very low
