# Supplementary material for: Incidence of sinus membrane perforation in transcrestal graftless maxillary sinus augmentation: a meta-analysis
Source: Acta Odontol Scand. 2026 Jun 4;85:46021. doi: 10.2340/aos.v85.46021 (PMC13241954; doi:10.2340/aos.v85.46021)

Supplementary material has been published as submitted. It has not been copyedited or typeset by Acta Odontologica Scandinavica.

Supplementary Figure 1 Forest plot —Complications.

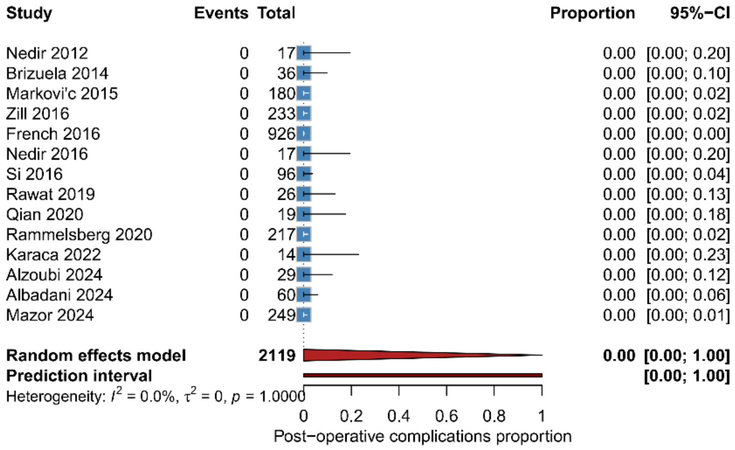

Supplementary Figure 2 Meta-regression analyses investigating sources of heterogeneity in membrane perforation rates. (A) Model A: Univariate analysis of Residual Bone Height (RBH). (B) Model B: Technique Categories. (C) Model C: RBH adjusted for Technique. (D) Model Comparison.

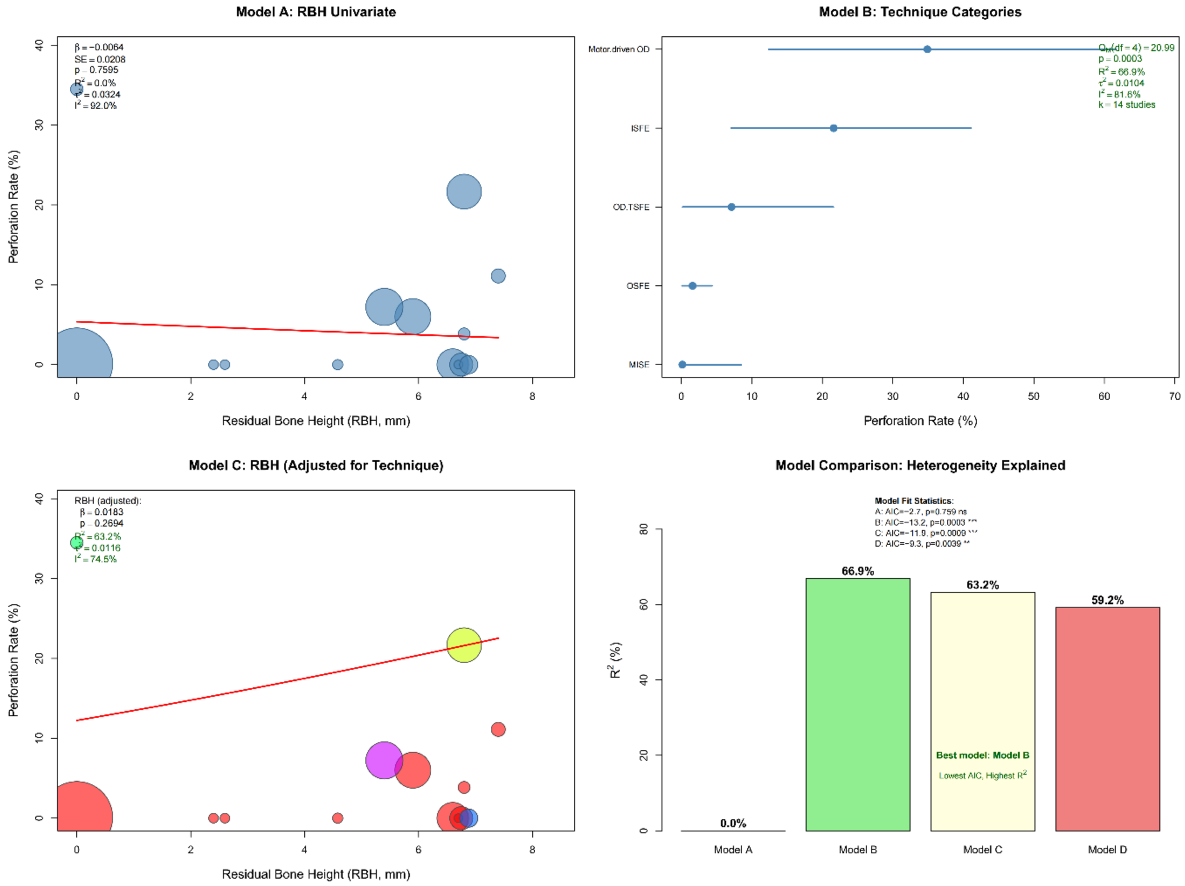

Supplementary Figure 3 Sensitivity analysis using the leave-one-out method.

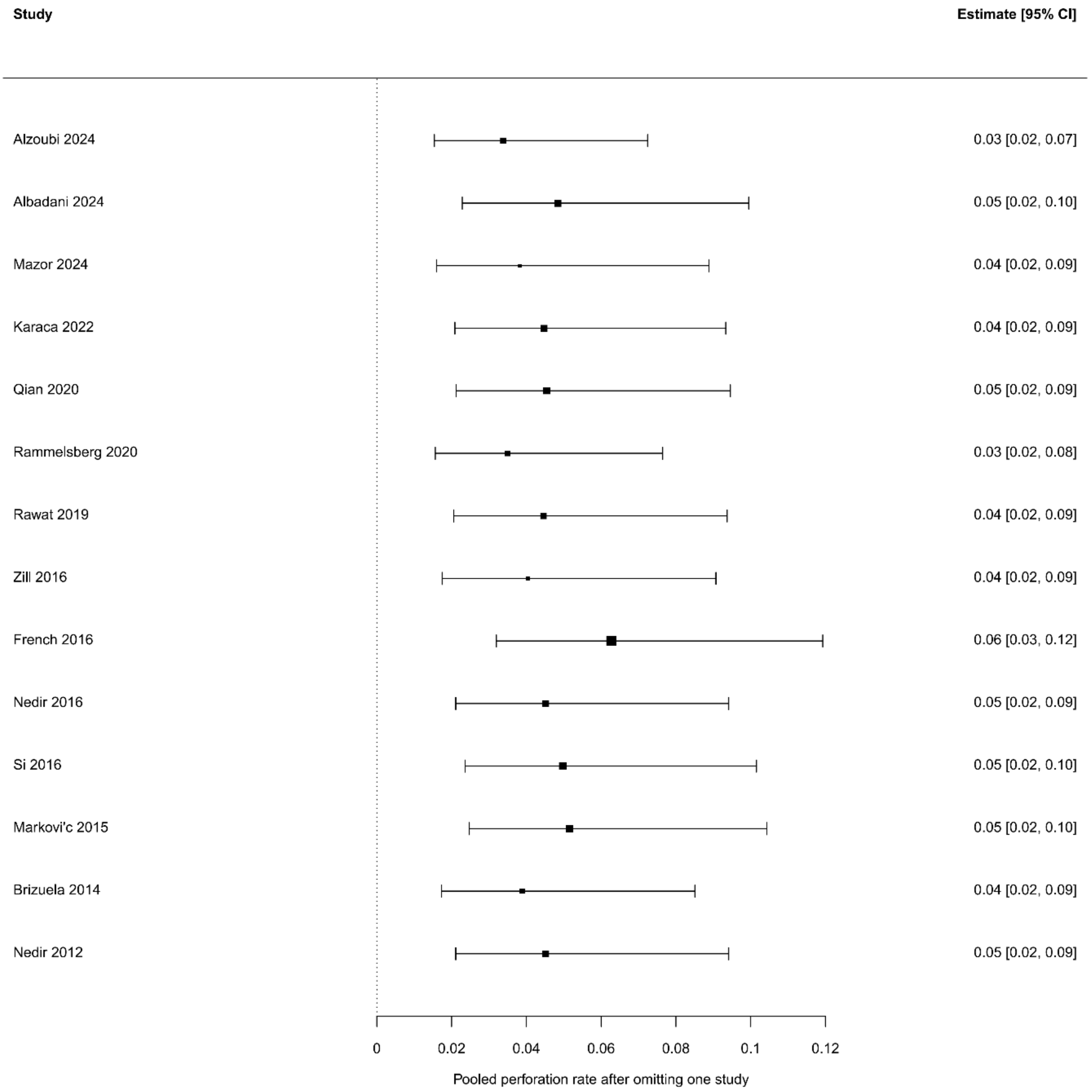

Supplementary Figure 4 Funnel plot assessing publication bias for the incidence of Schneiderian membrane perforation(A), early implant failure rate(B) and overall complications(C).

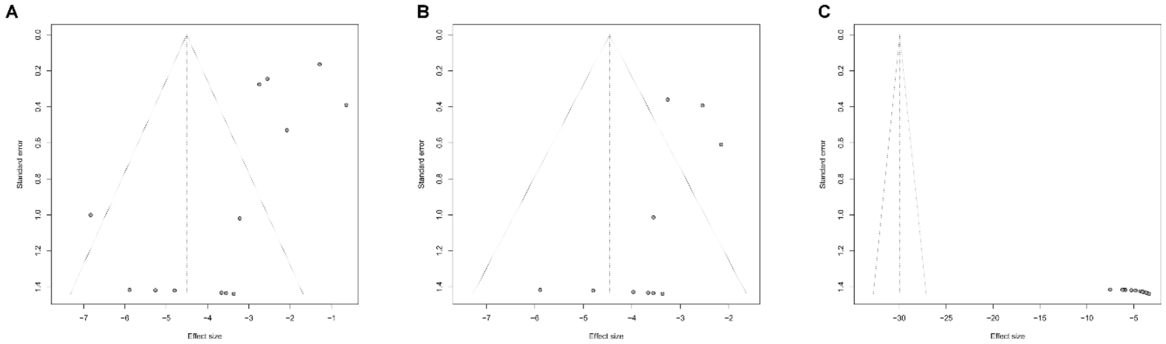

Supplement: Supplementary file 2 [file AOS-85-46021-s2.pdf]
